# Duodenal Ulcer Perforation Causing Acute Cholecystitis

**DOI:** 10.7759/cureus.61293

**Published:** 2024-05-29

**Authors:** Kensuke Konagaya, Nao Kume, Hidemitsu Ogino

**Affiliations:** 1 Surgery, Narita Tomisato Tokushukai Hospital, Chiba, JPN; 2 Vascular Surgery, Narita Tomisato Tokushukai Hospital, Chiba, JPN

**Keywords:** nsaids, fifth lumbar vertebra compression fracture, laparoscopic surgery, duodenal ulcer, acalculous cholecystitis

## Abstract

Acute cholecystitis is an inflammatory condition of the gallbladder, characterized by infection, ulceration, and neutrophilic infiltration of the gallbladder wall. Approximately 90% of cases are caused by gallstones. In contrast, acalculous cholecystitis is defined as the inflammation of the gallbladder in the absence of gallstones during diagnosis. The causes of acalculous cholecystitis include impaired blood flow to the gallbladder, chemical injury, bacterial or parasitic infections, and collagen vascular diseases. However, in this case, it was caused by an extremely rare condition: a duodenal ulcer penetration. Physical examination, blood tests, and ultrasound suggested a diagnosis of acute cholecystitis. However, contrast-enhanced CT showed no gallstones and revealed a partial mucosal defect in the first portion of the anterior duodenum. There was also wall thickening and increased density of the surrounding fat tissue, particularly around the gallbladder wall adjacent to the first portion of the anterior duodenum. Based on these findings, secondary cholecystitis due to perforation of a duodenal ulcer was diagnosed, and laparoscopic cholecystectomy with omental patching was performed. Although rare, a duodenal ulcer should be considered as a cause of acalculous cholecystitis.

## Introduction

Most cases of acute cholecystitis are triggered by the obstruction of gallstones at the neck of the gallbladder. This leads to bile stasis within the gallbladder and damage to the gallbladder mucosa, thereby activating inflammatory mediators and resulting in acute cholecystitis [1]. Although gallstones are the cause in 90% of cases, the remaining 10% are acalculous, with causes including trauma, surgery, shock, burns, infections, severe illnesses managed in the ICU, parenteral nutrition, and prolonged fasting [2-4]. Possible infectious agents include bacteria, fungi, parasites, and viruses, including cytomegalovirus, Epstein-Barr virus, and dengue virus [3,5]. There have also been reports of cases associated with COVID-19 infections since the onset of the pandemic [6]. We report a rare case of acalculous cholecystitis secondary to a duodenal ulcer. Although the gallbladder and duodenum are anatomically adjacent, the pathogenesis of cholecystitis and duodenal ulcers is fundamentally different, and they do not usually occur concurrently. Perforation and fistulation to other organs are occasionally reported as complications of duodenal ulcers; however, duodenal ulcer penetration causing cholecystitis is an extremely rare condition with a limited number of reported cases.

## Case presentation

A 72-year-old female, fully independent in daily activities, has a history of hypertension and dyslipidemia but takes no regular medication.

She presented to the hospital after a fall at home that resulted in limited mobility and was diagnosed with a fifth lumbar vertebra compression fracture. She was admitted for pain management and rehabilitation. On the 20th day of hospitalization, she suddenly experienced epigastric pain around 4 am. Her vital signs were within normal limits then. Physical examination revealed a soft abdomen with tenderness from the epigastric region to the right upper quadrant and a positive Murphy's sign.

Laboratory tests showed an elevated leukocyte count of 12,300/µL (normal range {NR}: 3,500-8,600/µL) with 84% neutrophils and hemoglobin of 11.3 g/dL (NR: 11.5-15.0 g/dL). Liver enzymes were normal: total bilirubin of 0.9 mg/dL (NR: 0.3-1.2 mg/dL), aspartate aminotransferase (AST) of 26 IU/L (NR: 7-38 IU/L), alanine transaminase (ALT) of 13 IU/L (NR: 4-44 IU/L), and alkaline phosphatase (ALP) of 78 IU/L (NR: 38-113 IU/L). Ultrasound examination showed mild thickening of the gallbladder wall without the dilation of the bile ducts. The ultrasound also indicated a positive sonographic Murphy's sign.

**Figure 1:**
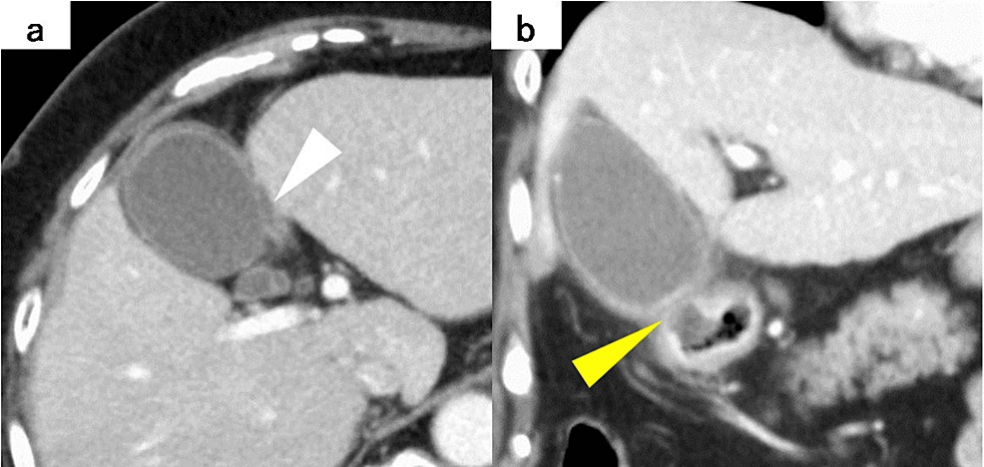
Contrast-enhanced CT Axial (a): Abdominal CT showed a thickened gallbladder wall and a small amount of pericholecystic fluid (white triangle). Coronal (b): There was a partial mucosal defect on the first portion of the anterior duodenum, suggestive of a duodenal ulcer (yellow triangle)

The contrast-enhanced CT revealed a partial mucosal erosion on the first portion of the anterior duodenum, accompanied by wall thickening and increased density of the surrounding adipose tissue, primarily around the adjacent gallbladder wall. No gallstones were identified, and the extrahepatic bile ducts were normal (Figure 1).

**Figure 2:**
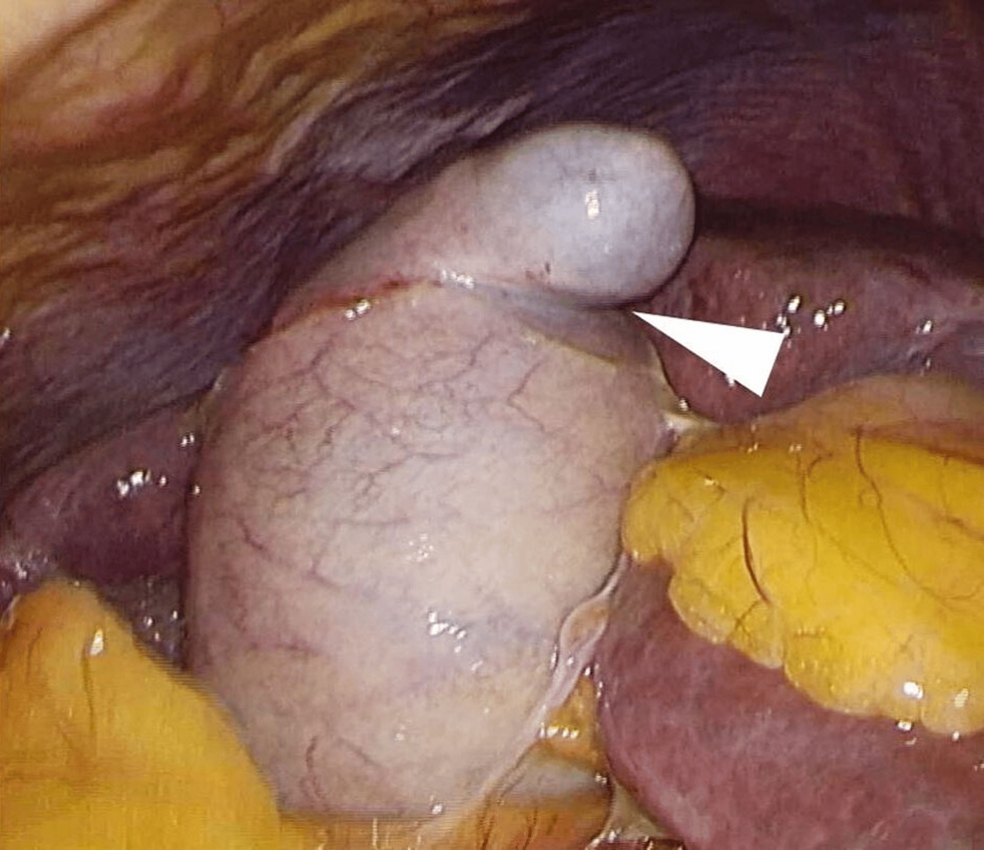
Phrygian cap The gallbladder fundus exhibited a localized kink (Phrygian cap) (white triangle)

**Figure 3:**
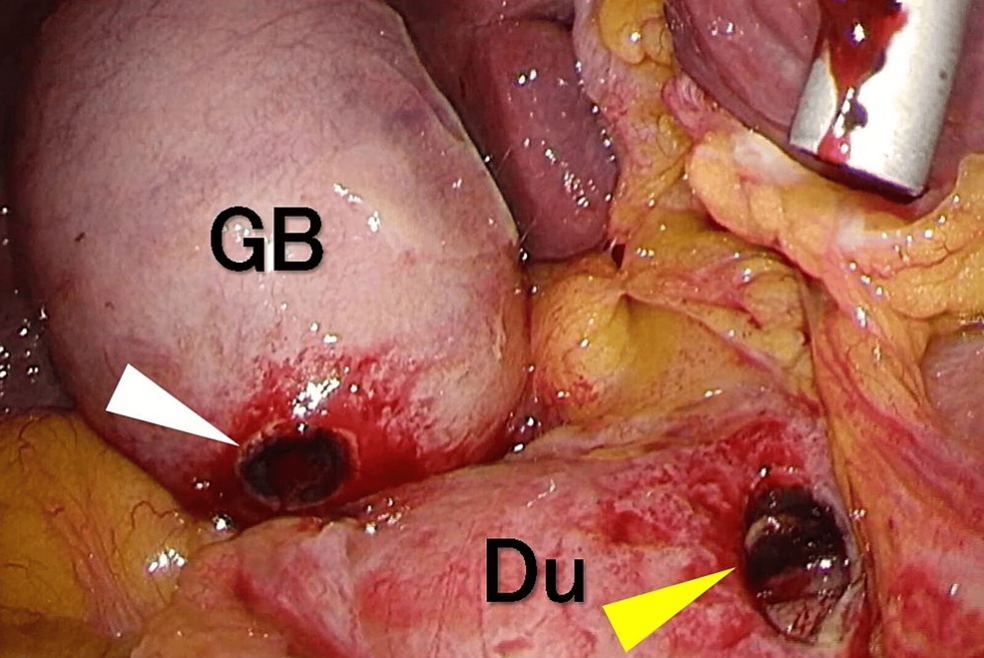
Complete view of both the gallbladder and duodenum The adhesion between the gallbladder and duodenal wall was easily lysed with blunt dissection. There was a first portion of duodenum with a perforated ulcer (yellow triangle) and a serosal defect on the gallbladder wall (white triangle) GB, gallbladder; Du, duodenum

The patient was diagnosed with acute cholecystitis due to duodenal ulcer penetration, necessitating emergency laparoscopic cholecystectomy. Upon port insertion and inspection of the abdominal cavity, there was no gallbladder distention or adhesion to the omentum. There were no signs of peritonitis such as ascites, pus around the gallbladder, or fibrin deposits. The gallbladder fundus exhibited a localized kink (Phrygian cap) (Figure 2), facilitating careful dissection between the gallbladder and the duodenum while cranially retracting the gallbladder. An approximately 15 mm perforation was noted on the first portion of the anterior duodenum. The gallbladder showed serosal defects but no perforation, and no bile leakage was observed during the operation (Figure 3). After performing a laparoscopic cholecystectomy, we performed a laparoscopic Graham patch repair, the most widely used technique for omentopexy for duodenal perforation. The operation took one hour and 20 minutes, with an estimated blood loss of about 30 mL.

**Figure 4:**
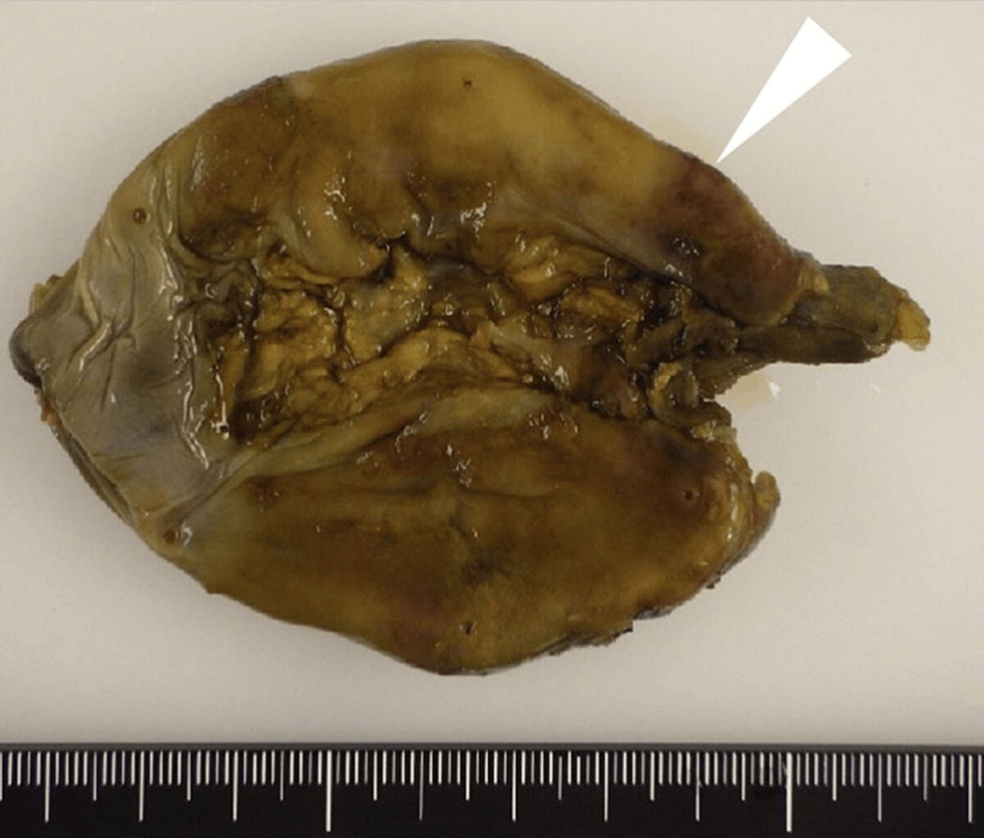
Macroscopic examination Macroscopic examination revealed visibly thickened gallbladder walls at the perforation site (white triangle). The mucosal surface was intact with no apparent perforation

Macroscopic examination revealed visibly thickened gallbladder walls at the penetration site. The mucosal surface was intact with no apparent perforation, and a moderate amount of green bile was present. No gallstones, biliary sludge, or sand was found (Figure 4).

**Figure 5:**
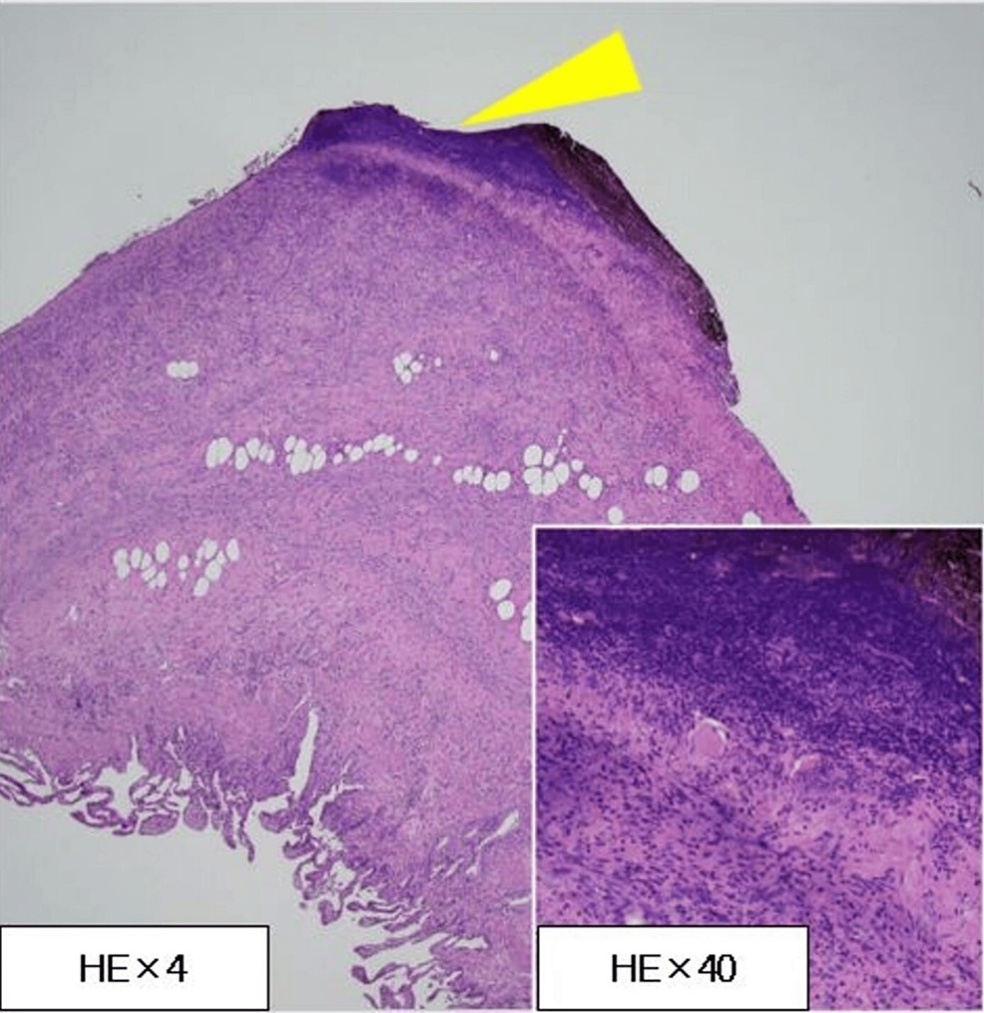
Histopathological examination Histopathological examination revealed inflammation at the gallbladder wall's perforation site (yellow triangle). There was no overt histopathological evidence of perforation H&E: hematoxylin and eosin

Histopathological examination revealed widespread inflammation at the gallbladder wall's penetration site, with marked infiltration of inflammatory cells on the serosal side. There was no overt histopathological evidence of perforation (Figure 5).

Postoperative tests for *Helicobacter pylori* immunoglobulin G (*H. pylori* IgG) antibodies showed negative results. Because nonsteroidal anti-inflammatory drugs (NSAIDs) were administered from the time of admission for the treatment of lumbar compression fractures and no proton pump inhibitors were used for ulcer prevention, NSAIDs were considered a likely cause of the duodenal ulcer. As a result, the pain medication was switched to acetaminophen. The postoperative course was smooth, with the patient quickly regaining appetite and resuming a normal diet within a few days. On postoperative day 10, the patient was transferred to a recovery rehabilitation ward and subsequently discharged home on day 30 post surgery.

## Discussion

Acute cholecystitis is characterized by the acute inflammation of the gallbladder, with 90% of cases caused by gallstones [7]. The remaining 10% are acalculous cholecystitis, which can result from trauma, surgery, shock, burns, infections, severe illnesses managed in the ICU, central venous nutrition, or prolonged fasting [2-4]. Duodenal ulcers are mucosal lesions caused by gastric acid, *Helicobacter pylori* infection, and nonsteroidal anti-inflammatory drugs (NSAIDs), with complications including perforation and penetration into other organs. The pancreas is affected in about 50% of penetrating ulcers, whereas the gallbladder is involved in approximately 2% of cases, making it extremely rare [8].

In this case, preoperative imaging revealed partial mucosal defects on the anterior wall of the duodenal bulb, as well as increased wall thickness and surrounding fatty tissue density around the adjacent gallbladder wall. There was no free intra-abdominal gas or abnormal gas formation within the gallbladder lumen, suggesting that although the duodenal ulcer had penetrated the gallbladder, it had not perforated, thus not forming a cholecystoduodenal fistula. Typically, the primary site of inflammation in cholecystitis is the gallbladder mucosa. However, histopathological examination in this case showed extensive inflammatory findings in the gallbladder wall, particularly around the penetration site, with severe inflammatory cell infiltration on the serosal side. This pathology raises the question of whether to classify such a condition as acute cholecystitis due to duodenal ulcer penetration, considering that the inflammation spread beyond the penetration site to involve the entire gallbladder, presenting an acute inflammatory change. Based on intraoperative and pathological findings, we diagnosed this unusual condition as secondary acute cholecystitis.

In a review of 819 cases by Waggoner and LeMone, cholecystoenteric fistulas accounted for the majority, with cholecystoduodenal fistulas comprising 51% [9]. Most cholecystoduodenal fistulas are caused by chronic inflammation due to gallstones or malignancy, with chronic cholecystitis accounting for about 90% of cases [10]. Reports of duodenal ulcers causing cholecystoduodenal fistulas are extremely rare. The gallbladder and duodenum are anatomically close, and both cholecystitis and duodenal ulcers are primary differential diagnoses for right upper quadrant pain. Although their etiologies differ significantly and mutual influence is rare, duodenal ulcer perforation should be considered a potential cause of acalculous cholecystitis.

In this case, intraoperative findings showed mild adhesions between the gallbladder and duodenum, which were easily separated with gentle dissection. This ease of dissection suggests that the adhesions were acute, resulting from duodenal ulcer penetration, rather than from infectious inflammatory changes or chronic inflammation due to cholecystitis or peritonitis.

Furthermore, the duodenal ulcer had not penetrated the entire gallbladder wall, as there was no bile leakage during surgery, and there were no signs of peritonitis, which made the laparoscopic procedure straightforward. Alexakis et al. evaluated 5,539 patients who underwent laparoscopic cholecystectomy over 10 years at a tertiary center in Greece, identifying seven patients with unsuspected perforated peptic ulcers. Of these, four were treated with laparoscopic suturing and omental patch repair, whereas the remaining three underwent open surgery [11].

Recently, laparoscopic surgery has been increasingly utilized for acute cholecystitis and duodenal ulcer perforation due to its minimally invasive nature. In this case, performing laparoscopic cholecystectomy and omental patch repair resulted in a favorable postoperative course, highlighting the benefits of minimally invasive laparoscopic surgery.

## Conclusions

Acute cholecystitis associated with gallstones is common, whereas acalculous cholecystitis is relatively infrequent. Among these cases, those in which a perforated duodenal ulcer causes gallbladder inflammation are even rarer. We anticipated this preoperatively and performed a laparoscopic cholecystectomy and omental patch repair. Given the anatomical proximity of the gallbladder and the duodenum, they can affect each other. Therefore, a perforated duodenal ulcer should be considered a potential cause of acalculous cholecystitis.
